# LEADOR: A Method for End-To-End Participatory Design of Autonomous Social Robots

**DOI:** 10.3389/frobt.2021.704119

**Published:** 2021-12-03

**Authors:** Katie Winkle, Emmanuel Senft, Séverin Lemaignan

**Affiliations:** ^1^ Division of Robotics, Perception and Learning, KTH Royal Institute of Technology, Stockholm, Sweden; ^2^ Department of Computer Sciences, University of Wisconsin–Madison, Madison, WI, United States; ^3^ Bristol Robotics Laboratory, University of the West of England, Bristol, United Kingdom

**Keywords:** social robotics, HRI, participatory design, mutual shaping of technology and society, autonomous robots, robot development

## Abstract

Participatory design (PD) has been used to good success in human-robot interaction (HRI) but typically remains limited to the early phases of development, with subsequent robot behaviours then being hardcoded by engineers or utilised in Wizard-of-Oz (WoZ) systems that rarely achieve autonomy. In this article, we present LEADOR (Led-by-Experts Automation and Design Of Robots), an *end-to-end* PD methodology for domain expert co-design, automation, and evaluation of social robot behaviour. This method starts with typical PD, working with the domain expert(s) to co-design the interaction specifications and state and action space of the robot. It then replaces the traditional offline programming or WoZ phase by an *in situ* and online teaching phase where the domain expert can live-program or teach the robot how to behave whilst being embedded in the interaction context. We point out that this live teaching phase can be best achieved by adding a learning component to a WoZ setup, which captures implicit knowledge of experts, as they intuitively respond to the dynamics of the situation. The robot then progressively learns an appropriate, expert-approved policy, ultimately leading to full autonomy, even in sensitive and/or ill-defined environments. However, LEADOR is agnostic to the exact technical approach used to facilitate this learning process. The extensive inclusion of the domain expert(s) in robot design represents established responsible innovation practice, lending credibility to the system both during the teaching phase and when operating autonomously. The combination of this expert inclusion with the focus on *in situ* development also means that LEADOR supports a mutual shaping approach to social robotics. We draw on two previously published, foundational works from which this (generalisable) methodology has been derived to demonstrate the feasibility and worth of this approach, provide concrete examples in its application, and identify limitations and opportunities when applying this framework in new environments.

## 1 Introduction

In the context of robotics research, participatory design (PD) attempts to empower non-roboticists such that they can shape the direction of robotics research and actively collaborate in robot design (Lee et al., 2017). Typically, PD is achieved by researchers running workshops or focus groups with end users and/or domain experts. Output may include potential use case scenarios (Jenkins and Draper, 2015), design guidelines/recommendations (Winkle et al., 2018), and/or prototype robot behaviours (Azenkot et al., 2016). Šabanović identified such methods as appropriate for the pursuit of a mutual shaping approach in robot design that is one that recognises the dynamic interactions between social robots and their context of use (Šabanović, 2010), an approach that we find compelling for designing effective and acceptable social robots efficiently. However, the automation of social robot behaviour, which requires a significant technical understanding of robotics and artificial intelligence (AI), is not typically considered during such activities.

Instead, common methods for the automation of social robot behaviour include utilising models based on human psychology (e.g., Theory of Mind, Lemaignan et al., 2017) or animal behaviour (Arkin et al., 2001) or attempting to observe and replicate human-human interaction behaviours (e.g., Sussenbach et al., 2014). This limits the potential for direct input from domain experts (teachers, therapists, etc.) who are skilled in the use of social interaction in complex scenarios. Previous work with such experts has demonstrated that a lot of the related expertise is intuitive and intangible, making it difficult to access in a way that can easily inform robot automation (Winkle et al., 2018). This is somewhat addressed by methods that capture domain expert operation of a robot directly, for example, end user programming tools (e.g., Leonardi et al., 2019) or learning from expert teleoperation of robots (e.g., Sequeira et al., 2016). However, these methods tend to focus on offline learning/programming. As such, there is no opportunity for experts to create an adequate, situated mental model of the capabilities of the robot, limiting the guarantee of appropriate behaviour when the robot is eventually deployed to interact with users autonomously.

Instead, we argue that robots should be automated by domain experts themselves, in real time, and whilst being situated in the interaction context; and that this automation should be done through a direct, bi-directional interaction between the expert and the robot. We refer to this as the *teaching phase*, where the robot is taught what to do by the domain expert, regardless of whether it is, e.g., a machine learning algorithm or an authoring tool that underpins this interaction. This live, *in situ*, and interactive approach allows *mutual shaping* to occur during robot automation, as the expert defines the program of the robot in response to the evolving dynamics of the social context into which the robot has been deployed.

### 1.1 Supporting a Mutual Shaping Approach to Robot Design

Šabanović proposed a *mutual shaping* approach to social robot design that is one that recognises the dynamic interactions between social robots and their context of use, in response to their finding that most roboticists were taking a technologically deterministic view of the interaction between robots and society (Šabanović, 2010). Studies of real-world human-robot interaction (HRI) motivate such an approach, because they demonstrate how mutual shaping effects impact robot effectiveness upon deployment in the real world. For example, the use and acceptance of robots in older adult health settings has been shown to be affected by situation and context of use factors such as user age and gender, household type, and the prompting of its use by others (Chang and Šabanović, 2015; de Graaf et al., 2015), i.e., factors unrelated to the functionality of the robot. The pursuit of a mutual shaping approach, primarily through use of PD and in-the-wild robot evaluation methods, gives the best possible chance of identifying and accounting for such factors during the design and development process, such that the robot has maximum positive impact on its eventual long-term deployment.

To this end, Šabanović describes four key practices that underpin a mutual shaping approach to support a “*socially robust understanding of technological development that enables the participation of multiple stakeholders and disciplines*”:

1) Evaluating robots in society: HRI studies and robot evaluations should be conducted “in the wild”, i.e., in the environments and context of use for which they are ultimately intended to be deployed (Ros et al., 2011).
2) Studying socio-technological ecologies: Robot design should be informed by systematic study of the context of use, and evaluation of robots should consider impact on the socio-technology ecology into which they have been deployed.
3) Outside-in design: Design constraints should be defined by empirical social research and the social context of use, rather than technical capabilities, and evaluation should be based on user experiences rather than internal measures of technical capability.
4) Designing with users: Stakeholders (those who will be directly affected by the deployment and use of the robot) should be included in identifying robot applications and thinking about how robots will be used and in designing the robot and its behaviour(s).

However, as we explain in Section 2, robot development at present typically represents a discontinuous process, particularly broken up by the automation of social robot behaviour. It still tends to heavily rely on technical expertise, executed in research/development environments rather than the real world, with little active inclusion of domain experts or other expert stakeholders. This discontinuity also represents a key hurdle to truly multi-disciplinary working, a disconnection between those of different academic backgrounds on the research team, which can result in a number of practical challenges and frustrations.

### 1.2 The *Led-By-Experts Automation and Design of Robots* Method

The generalisable method that we provide in this work is derived from two (independently undertaken) foundational works. First is the educational robot by Senft et al. (2019) for school children, in which a psychologist taught a robot to support children in an educational activity. After the teaching phase with 25 children, the robot was evaluated in further autonomous interaction with children, which demonstrated the opportunity of online teaching as a way to define autonomous robot behaviours.

Second is the robot fitness coach by Winkle et al. (2020). This work built upon the work by Senft et al. (2019) by integrating the online teaching method into an end-to-end PD process, whereby the same professional fitness instructor was involved in the co-design, automation, and evaluation of a robot fitness coach. This work also demonstrated the value of online teaching when compared to expert-designed heuristics as a next best alternative for defining autonomous robot behaviours with domain expert involvement. Both studies used a teaching phase where a domain expert interacted with the robot to create an interactive behaviour, and in both studies, the resulting autonomous robot behaviour was evaluated with success.

**FIGURE 1:**
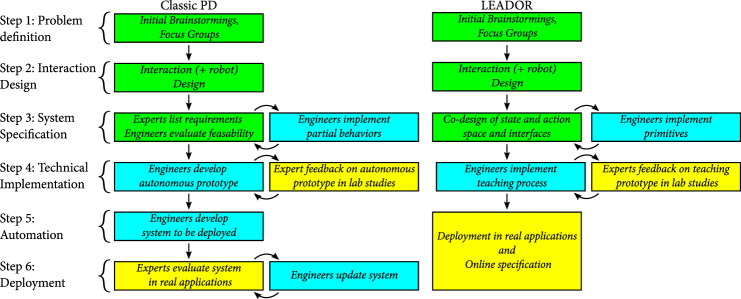
Comparison between a classic participatory design (PD) approach and LEADOR, our proposed end-to-end participatory design approach. Green activities represent joint work between domain experts, multidisciplinary researchers, and/or engineers; yellow activities are domain expert-led; blue activities are engineer-led. Compared to typical PD, the two key differences in our approach are the focus on developing a *teaching system* instead of a *final autonomous behaviour* in step 4, and the combining of autonomous action policy definition and deployment in the real world into a single step 5 + 6. In addition, our method reduces the amount of work that is carried out independently by engineers (i.e., with no domain expert or non-roboticist input).

From these works, we have derived a five-step, generalisable method for end-to-end PD of autonomous social robots (*Led-by-Experts Automation and Design Of Robots* or LEADOR), depicted alongside typical PD in Figure 1. The key stages of our approach, as referenced in the figure, can be summarised as follows:

1) Problem definition: *Initial brainstorming, studies of context of use, and studies with stakeholders.*
2) Interaction design: *Detailed refinement of robot application and interaction scenario, and choice/design of robot platform.*
3) System specification: *Co-design of the action space of the robot, input space, and teaching interface.*
4) Technical implementation: *Realisation of the third stage through technical implementation of underlying architecture and all sub-components and tools required for the teaching phase.*
5) Real-world deployment: *Robot is deployed in the real world, where a teaching phase is undertaken, led by the domain expert(s), to create autonomous robot behaviour.*

**FIGURE 2:**
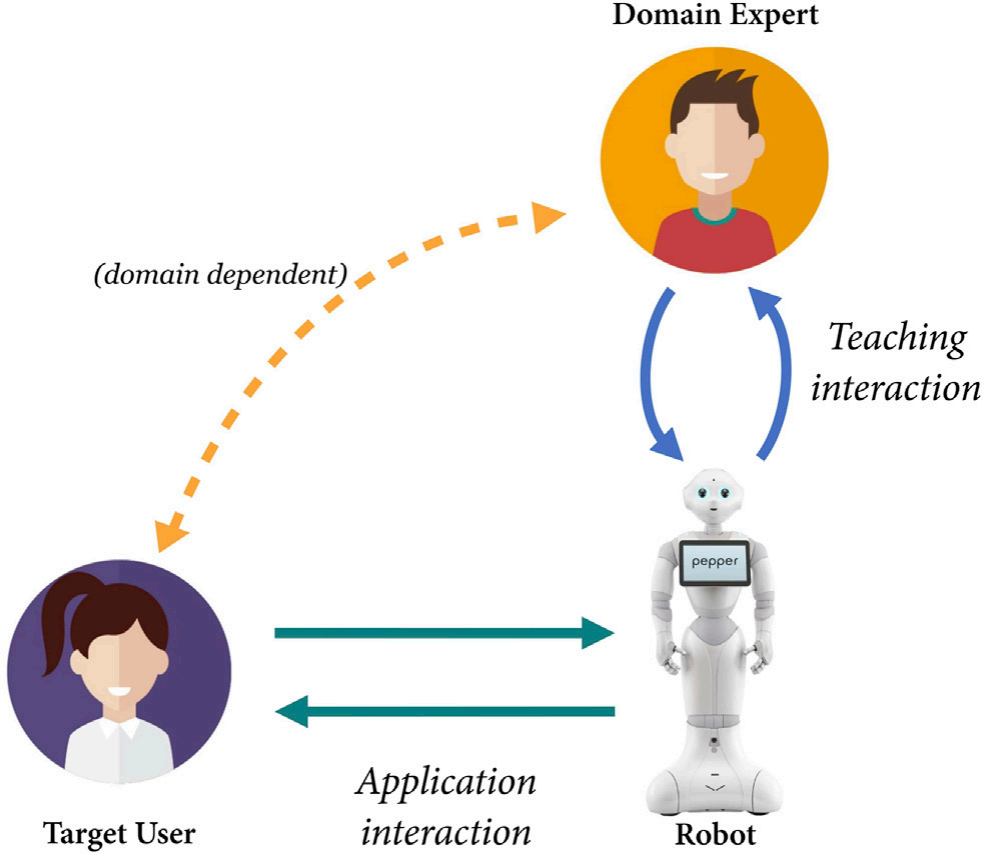
Three-way interaction between the domain expert, the robot, and the target user through which the expert teaches the robot during a teaching phase upon real-world deployment. Robot automation is therefore happening in the real world, whereas the robot is fully embedded in its long-term application context. The expert is teaching the robot through bi-directional communication, as the robot interacts with the target user. The extent of interaction(s) between the domain expert and target user should be consistent with what is envisaged for long-term deployment of the robot and is domain-dependent. People vector created by studiogstock - www.freepik.com.

The cornerstone of our method is to facilitate robot automation through direct interaction between the expert and the robot, during a “teaching phase”, whereby the domain expert teaches the robot what to do during real interaction(s) with the target user. The resultant interaction is depicted in Figure 2. Regardless of the specifics of the final interaction, the output of this phase is a robot that *can* operate autonomously but could also allow for continued expert-in-the-loop supervision and/or behaviour correction/additional training.

Through our foundational works, we demonstrate the flexibility in our method for developing autonomous robots for different long-term interaction settings. The educational robot by Senft et al. (2019) was intended for diadic, unsupervised robot-user interactions, whereas the robot fitness coach by Winkle et al. (2020) was intended primarily for diadic robot-user interactions but to be complimented with additional expert-user interactions/supervision and with additional expert-robot-user teaching interactions if necessary. LEADOR could also be used to design robots with other interaction requirements, e.g., an autonomous robot to be used in fully triadic expert-robot-user interactions or to facilitate permanent expert supervision and validation of autonomous behaviour.

In this paper, we have combined our experiences from these foundational works to propose an end-to-end PD process, centred around an *in situ* teaching phase, that uniquely delivers on the promises of mutual shaping and PD. We suggest that this approach is as *practical* as it is *responsible*, because our foundational studies demonstrate that we were able to create appropriate, intelligent, and autonomous social robot behaviour for complex application environments in a timely manner. As detailed in Senft et al. (2015) and Senft et al. (2019), this teaching phase is achieved by deploying the robot in the proposed use case and it is initially controlled completely by a human “teacher”. The teacher can progressively improve the robot behaviour *in situ* and generate a mental model of the policy of the robot. This teaching can continue until the domain expert is confident that the robot can satisfactorily operate autonomously. This approach therefore allows non-roboticist, domain experts to actively participate in creating autonomous robot behaviour. It also allows for the continual shaping of robot behaviour, because teaching can be seamlessly (re-)continued at any time to address any changes in the interaction dynamics, therefore better supporting a mutual shaping approach. We suggest that our methodology is particularly appropriate for use cases, in which difficult-to-automate and/or difficult-to-explain “intuitive” human domain expertise and experience are needed to inform personalised interaction and engagement (e.g., socially assistive robotics). The result then is an autonomous robot that has been designed, developed, and evaluated (by a multi-disciplinary research team) directly in conjunction with domain experts, within its real-world context of use, that can intelligently respond to complex social dynamics in ways that would have otherwise been very difficult to automate.

For clarity, hereafter, we use the term *domain expert* (or *teacher*) to refer to experts in an application domain. For example, these domain experts could be therapists, shop owners, or school teachers. These experts interact with the robot and specify its behaviour in a *teaching interaction* (even if no actual machine learning might be involved). On the other hand, *engineers* or developers refer to people with technical expertise in robotics or programming. They are the ones typically programming a robot behaviour or developing tools to be used by domain experts. Finally, the *target user* is the person a robot would interact with in the *application interaction*. For example, such target users could be children during a therapy session or store customers in a shopping interaction. This population is expected to interact with the robot at the point of use, rather than be the ones directly defining the autonomous robot behaviour.

## 2 Related Work

### 2.1 Participatory Design

PD is fundamentally concerned with involving the people who will use and/or will be affected by a technology in the design of that technology, with a focus on mutual learning between participants who typically represent either *domain experts* (users) or *technology experts* (designers) (Simonsen and Robertson, 2012). Contemporary PD has been concerned with combining typical, iterative PD practices with evaluation of the design in use under the concept of *sustained PD* or design as “emerging change” (Simonsen et al., 2010; Simonsen et al., 2014). Originally posed in the context of large-scale information systems projects, the sustained PD approach presented by Simonsen and Hertzum (2012) not only emphasises the evaluation of systems by exposing them to real-situated work practices but also notes associated challenges regarding management of a stepwise implementation process and the conducting of realistic, large-scale PD experiments.

This focus on implementation of the new technology as part of PD also therefore raises the notion of user participation in that implementation process. Fleron et al. (2012) demonstrated this in the context of first designing an electronic whiteboard with healthcare practitioners, followed by healthcare practitioner-led implementation of that whiteboard at two emergency departments. Notably, this work investigated differences in the experiences between staff who did and did not participate in this implementation process. Specifically, the participating staff (those responsible for the system implementation) identified some difficulties in understanding their role/responsibilities. The non-participating staff expressed a desire for earlier (pre-deployment) testing of the system but demonstrated positive “buy-in” of the system, nonetheless, with the authors positing this linked to reputed credibility of the system given it was (co-)developed and implemented by their peers. This points towards not only the potential benefits but also the challenges when trying to include end users and/or domain experts in this sustained PD process.

Specifically concerning PD, AI, and machine learning, Bratteteig and Verne (2018) note three key challenges when using PD with/for AI systems. First is that users and designers might struggle to conceptualise the possibilities and limitation of AI; second is that (machine learning–based) AI systems develop over time and hence are difficult to evaluate within a typical PD experimental period; and third is how to distinguish between “normal” use and training. Overall, the authors conclude that AI and machine learning can be part of a PD process, but that AI poses complex challenged that go beyond the scope of typical PD projects.

From the PD literature then, there is a clear motivation to explore PD processes that go beyond initial design to also include implementation and to understand how best to approach the PD of machine learning–based AI systems. The notion of using expert-in-the-loop machine learning for sustained PD that also includes implementation specifically sits at this exact intersection. However, whilst many contemporary PD works have described applications in the development and implementation of information technology systems, there seem to be very few (if any) that consider autonomous, social robot design, and development. Considering literature from the HRI field, however, a number of (interdisciplinary) HRI researchers have utilised and drawn from PD in the context of designing robots and their applications.

#### Participatory Design in Social Robotics

Here, we identify relevant works utilising PD and other related methodologies specifically in the context of designing social HRI. Notably, these works primarily originate from the HRI community, as opposed to the PD community, but most such works showcase one (or more) of the following methodologies:

1) *Ethnographic/”In-the-Wild” Studies*: These typically focus on understanding situated use and/or emergent behaviour(s) on deployment of a robot into the real world. Concerning robot design, such studies are inherently limited to the testing of prototypes, Wizard-of-Oz (WoZ) systems, or finished products (e.g., Forlizzi, 2007 and Chang and Šabanović, 2015). However, they might be used to inform initial design requirements (and their iteration) through observation of the use case environment and user behaviour.
2) *User-Centred Design (UCD)*: This aims to understand and incorporate user perspective and needs into robot design. Typically, researchers set the research agenda based on prior assumptions regarding the context of use and proposed robot application (e.g., Louie et al., 2014, Wu et al., 2012, and Beer et al., 2012).
3) *Participatory Design (PD)*: This encourages participants (users, stakeholders, etc.) to actively join in decision making processes which shape robot design and/or the direction of research. This typically involves participants having equal authority as the researchers and designers, with both engaging in a two-way exchange of knowledge and ideas (e.g., Azenkot et al., 2016 and Björling and Rose, 2019).

Lee et al. give a good overview of the above practices as employed in social robot and HRI design/research, with a particular focus on how the shortcomings of 1 and 2 can be addressed using PD (Lee et al., 2017). The authors use a case study of social robot PD from their own work to highlight a number of PD design principles for informing social robot design and further development of PD methodologies. They particularly highlight the empowering of PD participants to become active “robot co-designers” through *mutual learning*, as introduced previously, whereby there is a two-way exchange of knowledge and experience between researchers/designers and expert stakeholders. Through this process, users learn about, e.g., robot capabilities, such that they are better informed to contribute to discussions on potential applications, whilst the researchers/designers come to learn more about the realities of the proposed context of use from the perspective of the users.

Since publication of work of Lee et al., PD methods have been gaining visibility for the design of social robots, with other roboticists further refining PD methods and best practice for their use in social robotics and HRI. As such, PD works relating to ours can be grouped into two categories:

1) Results-focused publications that utilised PD methods.
2) Methodology-focused publications in which the authors share or reflect on PD methods for use in social robot/HRI design.

Works on 1) have typically taken the form of researchers working closely with prospective users and/or other stakeholders *via* focus groups, interviews, workshops, etc., with the researchers then concatenating their results to produce potential use case scenarios (Jenkins and Draper, 2015), design guidelines/recommendations (Winkle et al., 2018), and/or prototype robot behaviours (Azenkot et al., 2016). For example, Azenkot et al. (2016) used PD to generate specifications for a socially assistive robot for guiding blind people through a building. The study of the authors consisted of multiple sessions including interviews, a group workshop, and individual user-robot prototyping sessions. The initial interviews were used, in part, to brief participants about robot capabilities. The group session was used to develop a conceptual storyboard of robot use, identifying interactions between the robot guide and the user.

Winkle et al. (2018) conducted a study with therapists, utilising a novel focus group methodology combined with follow-up individual interviews to generate an expert-informed set of design guidelines for informing the design and use of socially assistive robots in rehabilitative therapies. The topic guides for each part of the study were designed to help the researchers to understand typical therapy practice and therapist best practices for improving patient engagement and to explore the ideas and opinions of the therapists on the potential role(s) social robots, which might play in rehabilitation. A key finding of this work was the extent to which intuitive, instantaneous behaviour of therapists is driven by situational factors specific to each individual client, making it difficult, for example, to extract any clear cut heuristics that might inform generalisable, autonomous social robot behaviour directly. The resultant design guidelines therefore suggested that socially assistive robots require “high-level” personalisation to each user as well as the ability to adapt, in real time, to, e.g., the performance of the user and other situational factors. This is one of the key works that motivates our effort to therefore facilitate expert-led, *in situ* robot teaching, to capture this sort of tacit social intelligence.

A follow up publication by the same authors then comes under category 2). Specifically, the authors provide more detail on their focus group methodology, and how it reflects a mutual shaping approach to social robot design, alongside a general guide in how it might be applied to other domains (Winkle et al., 2019a). The method combines elements of PD and UCD and utilises a demonstration of robot capabilities to support mutual learning between the researchers and participants. To evidence how this method supported mutual shaping in their work and why this was beneficial, the authors identify specific project-related considerations as well as new research directions that could only be identified in conjunction with their domain expert participants and also note that taking part in a focus group significantly (positively) impacted on the acceptance of participants of social robots.

Further, under category 2), Björling and Rose (2019) shared PD methods that they used in the context of taking an overall *human-centred design* approach to co-designing robots for improved mental health with teens. They present three method cases that cover novel and creative participatory techniques such as research design, script-writing and prototyping, and concluding with a set of PD principles for guiding design work with vulnerable participants in a human-centred domain. One of their methods revolved around inviting teens to act as WoZ robot operators. Specifically, their setup had one teen teleoperating a robot, whereas another teen recounted a (pre-scripted) stressful experience. In the second experiment, they utilised virtual reality (VR) such that one teen interacted, in an immersive VR environment, with a robot avatar teleoperated by a teen outside of that VR environment. From this study, the authors gathered data about the way teens collaborate and their perceptions of robot roles and behaviours. To this end, they demonstrated the value in expert (user) teleoperation of a proposed robot, not only to better understand both the use case requirements and user needs but also to generate exemplars of desirable autonomous robot behaviour. Alves-Oliveira et al. (2017) also demonstrated a similar use of puppeteering and role-play methods as part of a co-design study with children.

In summary, the work to date has demonstrated how PD methods can be used to study a proposed application domain in an attempt to ensure that researchers thoroughly understand the context of use and to elicit some expert knowledge for informing robot design and automation. This goes some way to supporting a mutual shaping and responsible robotics approach to social robot development. However, there remains two key disconnects in delivering truly end-to-end PD and mutual shaping in development of an autonomous social robot. First, robot automation is informed but not controlled or developed by domain experts. Second, there is a disconnection between this program definition and the real-world interaction requirements and situational specificities that will likely be crucial to overall robot success when deployed in real-world interaction.

### 2.2 Alternative Methods to Capture Domain Expert Knowledge

One of the key assumptions of PD in the context of robotics research is that the knowledge of the desired robot behaviour is held by domain experts and needs to be translated into programs. Typically, this translation is made by engineers, obtaining a number of heuristics from the domain experts and consequently automating the robot. Although widely applied even in PD research, this method only partially delivers on the promises of PD, because domains experts are used to inform robot behaviours but still rely on external actors (the engineers) to transform their intuition, knowledge, and expertise into actual code. Furthermore, this process can lead to a number of communication issues because members from different communities have different ways of expressing needs and desires. Nevertheless, there exist a number of alternative solutions to capture domain expert knowledge that could support a PD approach to robot automation.

#### 2.2.1 End User Programming Tools

A first solution is to create tools to allow domain experts to create robot behaviours themselves. The research on end user development or end user programming explores tools to allow domain experts or end users to create programs without requiring coding knowledge. Typical applications are home automation, application synchronisation (e.g., IFTTT or Microsoft Flow), or video games development. In addition, end user programming has seen large interest in robotics, for example, to create autonomous robot behaviours for both industrial robots (Paxton et al., 2017; Gao and Huang, 2019) and social robots (Leonardi et al., 2019; Louie and Nejat, 2020). These *authoring* tools are often developed by engineers and then provided to users to create their own applications without relying on text-based coding, for example, by using visual or block programming (Huang and Cakmak, 2017), tangible interfaces (Porfirio et al., 2021), or augmented reality (Cao et al., 2019).

However, whilst being more friendly for users, such methods still suffer from two main drawbacks. First, the interface is often developed by engineers without necessarily following principles of PD. Second, these methods often see the programming process as a discrete step leading to a static autonomous behaviour with limited opportunity to update the robot behaviour or little focus on testing and evaluating the created behaviour in real interactions. More precisely, users of these tools might not be the actual target of the application interaction and would program robots outside of the real context of use, forcing the aspiring developers to rely on their internal simulation of how the interaction should happen. For example, a shop owner could use an authoring tool to create a welcoming behaviour for a social robot and test it on themselves whilst developing the behaviour and then deploying it on real clients with limited safeguards. In such process, the developers have to use their best guess to figure out how people might interact with the robot and often have issues to infuse the robot with tacit knowledge, such as timing for actions or proxemics. This disconnect can lead to suboptimal robot behaviour as the robot will face edge cases in the real world that the designer might not have anticipated.

#### 2.2.2 Learning From Real-World Interaction(s)

A method to address this gap between an offline design phase and the real world is to mimic the expert whilst they perform the interaction. Using machine learning, systems can learn from the experts how robots should behave. For example, Liu et al. (2016) asked participants to role play an interaction between a shopkeeper and a client and recorded data about this interaction (e.g., location or speech of participants). From these recordings, Liu et al. learned a model of the shopkeeper, transferred it to the robot, and evaluated its HRIs. Similarly, Porfirio et al. (2019) recorded interaction traces between human actors and formalised them into finite state machine to create a robot behaviour. Whilst relying on simulated interactions, these methods provide more opportunities to developers to explore situations outside of their initial imagination.

One assumption of these methods is that robots should replicate human behaviour. Consequently, such methods allow the capture of implicit behaviours such as the timing and idiosyncrasies of human interactions. However, real-world interactions with robots might follow social norms different from ones between humans only. Consequently, learning directly from human-human interaction also presents limitations.

WoZ is an interaction paradigm widely used in robotics (Riek, 2012), whereby a robot is controlled by an expert deciding on what actions the robot should execute and when. The main advantage of this paradigm is to ensure that the robot behaviour is, at all times, appropriate to the current interaction. For this reason, WoZ has been extensively used in robot-assisted therapy and exploratory studies to explore how humans react to robots. Recent research has explored how this interaction can be used to collect data from real HRI and learn an appropriate robot behaviour. Knox et al. (2014) proposed the “Learning from the Wizard” paradigm, whereby a robot would first be controlled in a WoZ experiment used to acquire the demonstrations and then machine learning would be applied offline to define a policy. Sequeira et al. (2016) extended and applied this Learning from Demonstration (LfD), with an emphasis on the concept of “restricted-perception WoZ”, in which the wizard only has access to the same input space as the learning algorithm, thus reducing the problem of correspondence between the state and action spaces used by the wizard and the ones available to the robot controller. Both of these works could support a PD approach to robot automation, because they could be used to generate an autonomous robot action policy based on data from (non-roboticist) domain expert WoZ interactions in real-world environments. Nevertheless, the typical WoZ puppeteering setup results in an absence of interaction between the design/development team and the robot, which prevents designers from having a realistic mental model of the robot behaviour and does not allow for any mutual shaping between the wizard, the robot, and the contextual environment. Traditionally, LfD separates data collecting and learning into distinct steps, limiting the opportunity to know during the teleoperated data collection process at what point “enough” training data has actually been collected, because the system can only be evaluated once the learning process is complete. Similarly, when using end user programming methods, there is little opportunity to know how the system would actually behave when deployed in the real world. This lack of knowledge about the actual robot behaviour implies that robots have to be deployed to interact in the real world with limited guaranties or safeguards ensuring their behaviours are actually efficient in the desired interaction.

#### 2.2.3 Interactive Machine Learning

Interactive Machine Learning (IML) refers to a system learning online whilst it is being used (Fails and Olsen, 2003; Amershi et al., 2014). The premise of IML is to empower end users whilst reducing the iteration time between subsequent improvements of a learning system. Using IML to create robot behaviours through an interaction between a designer and an autonomous agent allows for full utilisation of the teaching skills of the expert. It has been shown that humans are skilled teachers who can react to the current performance of a learner and provide information specifically relevant to them (Bloom, 1984). Similarly, the previous research showed that this effect also exists, to a certain extent, when teaching robots. Using Socially Guided Machine Learning (Thomaz and Breazeal, 2008), a human teacher adapts their teaching strategy to robot behaviour and thus helps it to learn better. If able to observe (and correct) the autonomous behaviour of the robot, seeing the result of the robot behaviour as it progresses, then the expert can create a model of the knowledge, capabilities, and limitations of the robot. This understanding of the robot reduces the risk of over-trusting (both during training and/or autonomous operation) and introduces the potential for expert evaluation to become part of the verification and validation process.

## 3 A Blueprint for End-To-End Participatory Design

We identify the following requirements to extend PD into an *end-to-end* methodology that include the co-design of the automated behaviour of the robot. Such a method needs to allow for the following:

1) systematic observation and study of the use case environment in which the robot is to ultimately be deployed;
2) inclusion and empowerment of relevant stakeholders (users and domain experts) from the initial design phases, such that the design and application of the robot/interaction scenario is co-produced by researchers and stakeholders together;
3) (safe and responsible) evaluation of prototypes in the real-world environment(s) into which the robot is eventually intended to be deployed;
4) inclusion of relevant stakeholders in creation of autonomous robot behaviours, which should utilise interaction data collected in the real world;
5) two-way interaction between the domain expert “teacher” (e.g., a therapist) or designer and robot “learner” such that the teacher can better understand the state of the robot/to what extent learning “progress” is being made and hence adapt their teaching appropriately/flag any significant design issues; and
6) inclusion of relevant stakeholders (e.g., parents of a child in therapy) in (safe) evaluation of autonomous robot behaviours, as they perform in the real world.

Requirements 1 and 2 can be addressed by the typical PD methods discussed in Section 2.1.1, and requirements 3 and 6 can be addressed by carefully designed “in-the-wild” studies. In our work, we therefore look to specifically tackle requirements 4 and 5 by demonstrating how robot automation can be approached as an *in situ*, triadic interaction between domain expert teacher(s), robot learner, and target end user(s). With LEADOR, we showcase how this approach can be integrated into one continuous, end-to-end PD process that satisfies all of the above requirements.

Table 1 summarises the key outcomes of and some potential tools for each stage of LEADOR. Figure 1 shows how these steps compare to typical PD, as well as who (domain experts and/or engineers) are involved at each stage. Each stage is detailed in full below. Table 2 shows how these steps have been derived from/were represented in our two foundational studies.

**TABLE 1:** Key outcomes of and appropriate tools for each stage of LEADOR.

	Outcomes	Tools
1. Problem Definition	Domain understanding	Ethnographic studies, focus groups, brainstorming
2. Interaction Design	Interaction scenario, robot selection/design	Workshop, role-playing, low-tech prototyping
3. System Specification	State-action space for the robot, teaching tools	Brainstorming, behaviour prototyping
4. Technical Implementation	Robot system (sensors and actions), teaching system (authoring tools or learning algorithm)	Software development, laboratory studies, testing workshops
5. Real-World Deployment	Delivering on the application target, autonomous robot	*In situ* teaching by expert

**TABLE 2:** Overview of activities undertaken in the two case studies as exemplars for applying our generalised methodology. See Table 4 for a pictorial “storyboard” of this process and the co-design activities undertaken for development of the robot fitness coach.

	School-based educational robot	Gym-based robot fitness coach
Step 1	Decision by researchers based on experience to focus on learning food chain around an educational game for children of age 8–10	Researchers identified the NHS C25K exercise programme based on research goals (longitudinal, real-world HRI) but worked with a fitness instructor to observe typical environment and refine problem definition
Step 2	Decision by researchers to focus on robot-user interaction, with expert only providing robot commands and oversight of the robot behavior to ensure that each action is validated by them. Goal is to evaluate the creation of an autonomous robot	Decision in conjunction with the fitness instructor that the robot would lead exercise sessions (in which he would minimise interaction with exercisers) but that he would provide additional support (e.g., health advice, and stretching) outside of these. Goal is to create and demonstrate an effective, real-world SAR-based intervention *via* PD (as responsible robotics)
Step 3	Using SPARC (Senft et al., 2015) as interaction framework, robot state, and action spaces defined by researchers. Teaching through a GUI on a tablet	Also using SPARC (Senft et al., 2015) the robot state and action spaces as well as the teaching GUI were all co-designed with the fitness instructor
Step 4	Implementation of all the actions and learning algorithm. Prototype evaluation in laboratory. Initial pilot study in schools for evaluating the game with the target population and used as teacher training	Implementation of all the actions and learning algorithm. Fitness instructor provided all dialogues for robot actions. Prototype evaluation was undertaken in the laboratory, and in the final study, gym environment, final robot placement, and system installation details were also decided in conjunction with the fitness instructor
Step 5	Deployment in two local schools with more than 100 children over multiple months. Between-subject evaluation with three conditions: a passive robot, a supervised robot (during the teaching interaction) and an autonomous unsupervised robot	Deployed in to the university gym for teaching and autonomous evaluation through delivery of the C25K programme (27 sessions over 9–12 weeks) to 10 participants. Ran a total of 232 exercise sessions of which 151 were used for teaching the IML system, 32 were used for evaluating the IML system when allowed to run autonomously and 49 were used to test a heuristic-based “control” condition (all testing was within-subject)

### 3.1 Step 1: Problem Definition

As noted in Figure 1, Step 1 of our method aligns to best practice use of PD as previously demonstrated in social robotics. The purpose of this stage is to generate a thorough understanding of the use context in which the robot is to be deployed and to invite stakeholders to influence and shape the proposed application. It would likely include observations, focus groups, and/or interviews with a variety of stakeholders.

The focus group methodology presented in Winkle et al. (2019a) is one appropriate method that could be used for engaging with stakeholders at this stage because it facilitates expert establishment of non-roboticists, broad discussion of the application context (without presentation of a pre-defined research agenda), participant reflection on the context of use “as is”, and researcher-led sharing of technical expertise, followed by detailed consideration and refinement of the research agenda based on researchers and participants now being equal co-designers.

### 3.2 Step 2: Interaction Design

Similarly to Step 1, Step 2 of our method also aligns to best practice use of PD as previously demonstrated in social robotics. The purpose of this stage is to define and refine the interaction scenario(s) that the proposed robot will engage in and hence the functionalities/capabilities that it might require. The robot platform should also be chosen at this stage. For simplicity, here, we have equated robot platform *choice* with robot platform *design*. Much current social robotics research utilises off-the-shelf robot platforms (e.g., Pepper and NAO from Softbank Robotics), but others focus on the design of new and/or application-specific platforms. Either can be appropriate for LEADOR as long as the choice/design is participatory with stakeholders (for a good example of PD in design of a novel robot, see the work of Alves-Oliveira et al. (2017) on designing the YOLO robot).

Focusing then on more specific application of the robot and the interaction(s) that it should engage in, the methods for PD might include focus groups similarly as those in Step 1 but could also include more novel and/or creative PD activities such as script writing (Björling and Rose, 2019), role playing (including also stakeholder teleoperation of the robot) (Björling and Rose, 2019; Alves-Oliveira et al., 2017), and accessible, “low-tech” prototyping (Valencia et al., 2021).

Note that there is an important interaction design decision to be made here regarding what final deployment of the robot “looks like” in terms of long-term oversight by/presence of domain expert(s) (those involved in its co-design or otherwise) and the role those experts play with regard to the target user. This can be reflected in the teaching interaction setup, specifically with regard to the amount of interaction between the domain expert(s) and target users (see Figure 2). For example, it was decided early on in the design of fitness coach robot by Winkle et al. (2020) that there was no intention to ever fully remove the expert presence from the interaction environment. As an alternative, in the work of Senft et al. (2019), the intention from the onset was to create a fully autonomous and independent robot that interacted alone with the target users. Such decisions regarding the role of domain experts would ultimately emerge (explicitly or implicitly) in conjunction with deciding the functionalities of the robot and the further system specification undertaken in Step 3. However, this long-term desired role of the domain expert(s) should be made clear, explicitly, at this stage, such that it can be reflected in the approach to program definition.

### 3.3 Step 3: System Specification

As shown in Figure 1, it is at this stage that our method begins to diverge from the typical PD process, although we continue to utilise PD methods. This step is concerned with co-design of system specifities required to 1) deliver the interaction design resulting from Step 2 and 2) facilitate expert-led teaching phase on real-world deployment that is fundamental to our method (see Step 5). In summary, the aim of this step is to co-design the action space and input space of the robot and the tool(s) that are required to facilitate the bi-directional teaching interaction between the domain expert and the robot. There is also some similarity here to the design process for a WoZ or teleoperated system, which would also require design of the action space of the robot and an interface for (non-roboticist) teleoperation of the robot. The key difference here is the additional requirement to specify the input space of the robot and the choice of teaching tools for the move towards autonomy during Step 5.

### 3.4 Step 4: Technical Implementation

The main development effort for our method lies in producing the full architecture and tools to allow domain experts to specify autonomous robot behaviour. We note here that the technical implementation required is likely to be greater than that required for a typical WoZ setup and might not be simpler than heuristics-based robot controller.

Four main components need to be developed during this phase:

1) Set of high-level actions for the robot;
2) Set of sensory inputs that will be used to drive the future robot behaviour;
3) A representation of the program which will encode autonomous behaviour; and
4) Expert tools to specify the mapping between the sensory state and the actions.

With our method, the program representation could take the shape of a machine learning algorithm taking inputs from the expert *via* the interface and learning a mapping between the current state of the world when the action was selected and the action itself (the approach taken in our foundational works). Alternatively, the representation could allow the expert to encode a program explicitly, for example, through state machines or trigger-action programming, whilst allowing the expert to update the program in real time and to control the robot actions to ensure that they are constantly appropriate.

A typical automation system would replace the expert tools with an actual definition of the behaviour making use of the program representation to map sensors to actions and define fully an autonomous behaviour. On the other end of the spectrum, a WoZ setup might not need a representation of the program but instead would rely on the interface to display relevant sensory inputs to the wizard (if any) and allow them to select what action to do.

### 3.5 Step 5: Real-World Deployment and Teaching Phase

Undertaking robot automation (and evaluation) in-the-wild is a key part of LEADOR. To satisfy requirements 5 and 6 as laid out in the introduction, support a mutual shaping approach to robot design, and ensure appropriate robot behaviour, the *teaching phase* should adhere to the following:

1) It must be undertaken *in situ*, i.e., in the context of the final context of use, and with the real target population.
2) It must utilise a domain expert teaching the robot as it delivers on the application interaction.
3) The expert-robot interaction should be bi-directional, i.e., the expert should be able to define and/or refine the autonomous behaviour policy of the robot, whereas the robot informs the expert about its status.

Requirement 1 ensures that the approach is ecologically valid and that the information used by the expert for the automation are suited to the real challenges and idiosyncrasies of the desired context of use.

Requirement 2 ensures that people with domain knowledge can encode that knowledge in the robot. Furthermore, the presence of the expert should be used to ensure that the robot is expressing an appropriate behaviour at all times. As the teaching happens in the real world, with the real users, there is limited space for trial and error. The expert can be used as a safeguard to ensure appropriate robot behaviour even in the initial phases of the teaching.

Requirement 3 ensures that the expert can create a mental model of the robot behaviour. This point is a key difference to non-interactive teaching methods such as the ones based on offline learning (e.g., Sequeira et al., 2016). With the feedback of the robot on its policy (through suggestions or visual behaviour representation), the expert can assess the (evolving) capabilities of the robot and decide what inputs would further improve the policy of the robot.

Finally, during this real-world deployment, if the robot is ultimately expected to interact autonomously/unsupervised, then the expert can use their mental model of the robot behaviour to decide when enough teaching has been done and when the robot is ready to interact autonomously. By relying on online teaching, this decision does not have to be final because the expert could seamlessly step back into the teacher position when the robot interacts with sensitive populations or if the robot requires additional refinement of its policy.

## 4 Foundational Studies

The LEADOR method is primarily derived from two foundational studies made by the authors, which were themselves informed by the previous experiences of authors working with domain experts in the design of social robots. The first one, presented in Senft et al. (2019), explores a study with 75 children on how the teaching interaction could be used to create an autonomous robot behaviour. As shown in Table 2, this study did not employ PD, the authors (researchers in HRI) did the early steps by themselves based on their previous related experiences. The second one, presented in Winkle et al. (2020), built on the first study by utilising the same teaching approach to robot automation but incorporating that into an end-to-end PD process to support mutual shaping. The end goal of each study was also slightly different, as Senft et al. (2019) aimed to produce a robot that would ultimately interact with users with little to no further expert involvement. Winkle et al. (2020) also aimed to produce an autonomous robot that would primarily interact 1:1 with users, but with no desire to remove the expert, who would have their own interactions with the users, and/or provide additional teaching to the robot should they deem it necessary.

### 4.1 Study 1: Evaluating the Teaching Interaction

The goal of this first study was to evaluate if the teaching interaction could be used to create autonomous social behaviours (Senft et al., 2019). This study was designed by the authors, who had experience designing robots for the application domain but did not involve external stakeholders such as teachers.

During the problem definition phase, researchers decided to contextualize the work in robot tutoring for children and explore questions such as how robots can provide appropriate comments to children (both in term of context and time) to stimulate learning. This work was based on experience and knowledge from the researchers about educational robotics.

During the interaction design phase, researchers decided to focus the application interaction around an educational game where children could move animals on a screen and understand food nets. This part included an initial prototype of the game. As the goal was to explore how autonomous behaviours could be created, the teacher was not involved in the game activity, and only the robot was interacting with the child. The robot used was a NAO robot from Softbank Robotics.

In the system specification, the state and action spaces of the interaction were selected. Examples of state include game-related component (e.g., distance between animal) and social dynamics elements (e.g., timing since last action of each agents). The actions of the robot were divided into five categories: encouragements, congratulations, hints, drawing attention, and reminding rules. The teacher-robot interaction used SPARC (Senft et al., 2015).

In the technical implementation phase, the learning algorithm was developed, tested, and interfaced with the other elements of the system. The teaching interface was also created in such a way as to allow the teacher to select actions for the robot to execute and receive suggestions from the robot. At this stage, initial prototypes were tested in laboratory studies and schools.

In the real-world deployment, authors evaluated the system in two different schools with 75 children. The study adopted a between-participant design and explored three conditions: a passive robot, a supervised robot (referring to the teaching interaction), and an autonomous robot (where the teacher was removed from the interaction and the learning algorithm disabled).

Results from the study showed that the teaching interaction allowed the teacher to provide demonstrations to the robot to support learning in the real world. The teacher used the teaching interaction to create a mental model of the robot behaviour. When deployed to interact autonomously, the robot enacted a policy presenting similarities with the one used by the teacher in the teaching phase: the frequency of actions was similar and the robot captured relation and timing between specific events and actions (e.g., a congratulation action should normally be executed around 2 s after an eating event from the actions of a child). Overall, this study demonstrated that human can teach robot social policy from *in situ* guidance.

### 4.2 Study 2: Teaching Interaction as Participatory Design

The goal of this study was to use the teaching interaction approach to facilitate creation of a fully expert-informed/expert-in-the-loop autonomous socially assistive robot-based intervention for the real world. The fundamental activity to be delivered by robot, the NHS C25K programme, was selected by the researchers based on this research goal, but all study implementation details were decided and designed in conjunction with a domain expert (fitness instructor) throughout. Given the end-to-end and constant expert involvement for this study, there was seamless progression and some overlap between the problem definition, interaction design, and system specification phases, as we present them for LEADOR. A number of co-design activities were undertaken (over a total of six sessions totalling approximately 12.5 h), which ultimately covered all of these key phases, sometimes in parallel, allowing for iteration of the overall study design.

Problem definition was achieved by researchers working with the fitness instructor to 1) understand how a programme like C25K would be delivered by a (human) fitness instructor and 2) explore the potential role a social robot might take in supporting such an intervention. This involved the researchers visiting the university gym and undertaking mock exercise sessions with the instructor, and the instructor visiting the robotics laboratory to see demonstrations of the proposed robot platform and a presentation by the researchers on their previous works and project goals. The robot used was a Pepper robot from Softbank Robotics.

For the interaction design, the researchers and fitness instructor agreed that exercise sessions would be led by the robot and primarily represent robot-user interactions, with the fitness instructor supervising from a distance and only interacting to ensure safety (e.g., in the case of over exertion). As this study also aimed to test (within-subject) the appropriateness of resultant autonomous behaviours, it was decided to purposefully leave the details of the role of the fitness instructor somewhat ambiguous to exercising participants. The instructor was not hidden away during the interaction, and it was clear he was supervising the overall study, but exercisers were not aware of the extent to which he was or was not engaging in teaching interactions with the robot during sessions. As noted in Section 3, deciding on what long-term deployment should “look like” in terms of robot-user-expert interactions is a key design requirement at this stage. For the robot fitness coach, we imagined a “far future” scenario, where one of our robot fitness coaches would be installed next to every treadmill on a gym floor, supervised by one human fitness instructor. That instructor would ensure the physical safety of exercisers and still play a role in their motivation and engagement as human-human interaction is known to do. This type of interaction with one expert, multiple robots, and multiple target users is a common goal in many assistive robot applications where some tasks could be automated, but there is a desire to keep an expert presence to, e.g., maintain important human-human interactions and ensure user safety.

The system specification represented somewhat of a “negotiation” between the researchers and the fitness instructor, as he identified the kind of high level action and inputs he felt the robot ought to have, and the researchers identified how feasible that might be for technical implementation. The state space consisted of static and dynamic features that were designed to capture exerciser engagement, task performance, and motivation/personality, all identified by the fitness instructor as being relevant to his decisions in undertaking fitness instruction himself and hence teaching the robot how best to interact with a particular participant. The action space was divided into two categories: task actions and social supporting actions. The task actions were fundamentally set by the C25K programme (i.e., when to run or walk and for how long at a time). The social supporting actions were then broken down into eight sub-categories covering time reminders, social interaction, performance feedback, praise, checking on the user, robot animation, and two proxemics-related actions (leaning towards/away from the user). Importantly, system specification for this study also included co-designing the GUI that would facilitate the bi-directional teaching interaction (also utilising SPARC, Senft et al., 2015) between the robot and the fitness instructor with the fitness instructor himself.

The technical implementation phase essentially mirrored that of Study 1: the learning algorithm was developed, tested, and interfaced with the other elements of the system. The teaching interface was also finalised based on the co-design activities described previously and similarly allowed the fitness instructor to select actions for the robot and to respond to its suggestions. Initial prototypes of both the robot and the GUI were tested in the laboratory studies and the final gym environment.

In the real-world deployment, researchers evaluated the system in a university gym with 10 participants recruited to undertake the 27-session C25K programme over a maximum of 12 weeks. The study adopted a within-subject design and explored three conditions: a supervised robot (referring to the teaching interaction), an autonomous robot (where the fitness instructor was still in position but allowed all learner-suggested actions to auto-execute), and a heuristic-based autonomous robot; a “control” condition for comparing the “teaching interaction as PD” approach to, representing a “next best” alternative for generating expert-informed autonomous behaviour.

Results from the study again demonstrated the feasibility of SPARC and IML for generating autonomous socially assistive robot behaviour suggested that the expert-robot teaching interaction approach can have a positive impact on robot acceptability (by the domain expert and targets users) and that the teaching approach yields better autonomous behaviour that expert informed heuristics as a “next best” alternative for expert-informed autonomous behaviour creation.

### 4.3 Evidence of Mutual Shaping

Typical PD facilitates mutual shaping as it allows non-roboticist, domain experts to shape research goals, design guidelines, and evaluate robot prototypes, etc. Here, we reflect on observations of mutual shaping effects in our foundational works, specifically resulting from our teaching approach to robot automation.

During our first study, we observed evidences of mutual shaping and the teacher creating a mental model of the robot. For example, our teacher realised with experience that children tended have issues with some aspect of the game (i.e., what food a dragonfly eats). Consequently, she changed her strategy to provide additional examples and support for this aspect of the game. Similarly, the teacher also found that the robot was not initiating some actions often and consequently used these actions more frequently towards the end of the teaching phase to ensure that the robot would exhibit enough of these actions. This exactly evidences the notion that human teachers can tailor their teaching to the progress of a (robot) learner (Bloom, 1984; Thomaz and Breazeal, 2008).

In the second study, we were able to demonstrate mutual shaping in the way the fitness instructor used the robot differently for different participants and/or at different stages of the C25K programme. The longitudinal nature of this study, combined with our approach in supplementing the diadic robot-user interactions with expert-user interactions, meant that the fitness instructor got to know exercise styles/needs of each user and could tailor the behaviour of the robot accordingly. This resulted in the autonomous robot similarly producing behaviour that varied across participants. Similarly, as the programme progressed, the fitness instructor could tailor the behaviour of the robot to reflect the changing exercise demands (e.g., using fewer actions when the periods of running were longer). The flexibility of our approach was also demonstrated when, in response to this increase in intensity, the fitness instructor requested that we add a robot-led cool-down period to the end of each exercise session. This was relatively simple to implement from a technical perspective (an additional “walk” instruction at the end of each session plan) but represented a new part of the session for which there existed no previous training data. As we made this change within the teaching phase (before the switch to autonomous operation), the instructor was able to address this, such that the robot was able to successfully and appropriately support this new cool-down phase when running autonomously.

We also saw an interesting, emergent synergy in the way that the fitness instructor utilised and worked alongside the robot coach. Towards the end of the study, as exercise sessions became more demanding, the fitness instructor took more time at the end of each session to undertake stretching exercises with each participant. This leads to small amounts of overlap between each participant, at which point the fitness instructor would start the next participant warming up with the robot, whilst he finished stretching with the previous participant. We find this to be compelling evidence of the way domain experts will change their practice and/or the way they utilise technological tools deployed into their workplace, particularly when they can be confident in their expectations of how that technology will perform, as is particularly fostered by our approach.

### 4.4 Interactive Machine Learning for the Teaching Interaction: Opportunities and Limitations

As noted previously, both of our foundational studies utilised IML *via* the SPARC paradigm to facilitate the teaching interaction. From a technical perspective, our foundational studies demonstrate the feasibility and relative effectiveness (in terms of teaching time) of this approach. Fundamentally, LEADOR is agnostic with regard to the specific computational approach to facilitating the teaching interaction, but we find IML to be a particularly compelling solution, in line with the overall aims of the method, as it makes for an intuitive bi-directional teaching interaction for the domain expert. Specifically, through one single interface, they can see what the robot intends to do (and potentially why) before that action is executed, improving their understanding of the learning progress of the robot, and instantiate teaching exemplars in real time, informed by that understanding as well as the instantaneous requirements of the application task.

However, here, we draw attention to one key limitation regarding expert-robot interactions and assessment when using IML. An important element of mutual shaping not considered here is if/how/to what extent the suggestions made by the learning robots may have influenced the domain experts. For example, had the learning robots not been making suggestions, such that the robot was entirely controlled/teleoperated by the experts, would the action distribution and timing of actions remained the same? Further, if the experts did not have the ability to actively reject suggestions (indicating that the learner was not producing appropriate robot behaviour), then would they still have *post hoc* identified those actions as being inappropriate?

This is particularly interesting given the high number of suggested actions still being rejected at the end of the training phase, in both of our foundational studies, immediately followed by seemingly appropriate robot behaviour that was positively evaluated by the experts themselves during autonomous operation. Success of our approach inherently assumes that the domain expert/system “teacher” would provide a “correct” and fairly consistent response; i.e., that they 1) can correctly assess the quality of each action suggested by the robot and make an informed about whether this action should be executed and 2) are always able to ensure that required robot actions are executed in a timely fashion. With SPARC, these robot suggestions are the main means to help the expert create a mental model of the robot behaviour. Consequently, whilst our results demonstrate that the IML does fundamentally “work” for automating robot behaviour and that our domain experts did construct a mental model of the behaviour of the robots, there remains an open question regarding how the robot could improve the transparency of its behaviour to actively support mental model creation for the teacher.

## 5 Discussion

### 5.1 A Flexible and Effective Method for Automating Social Robots

**FIGURE 3:**
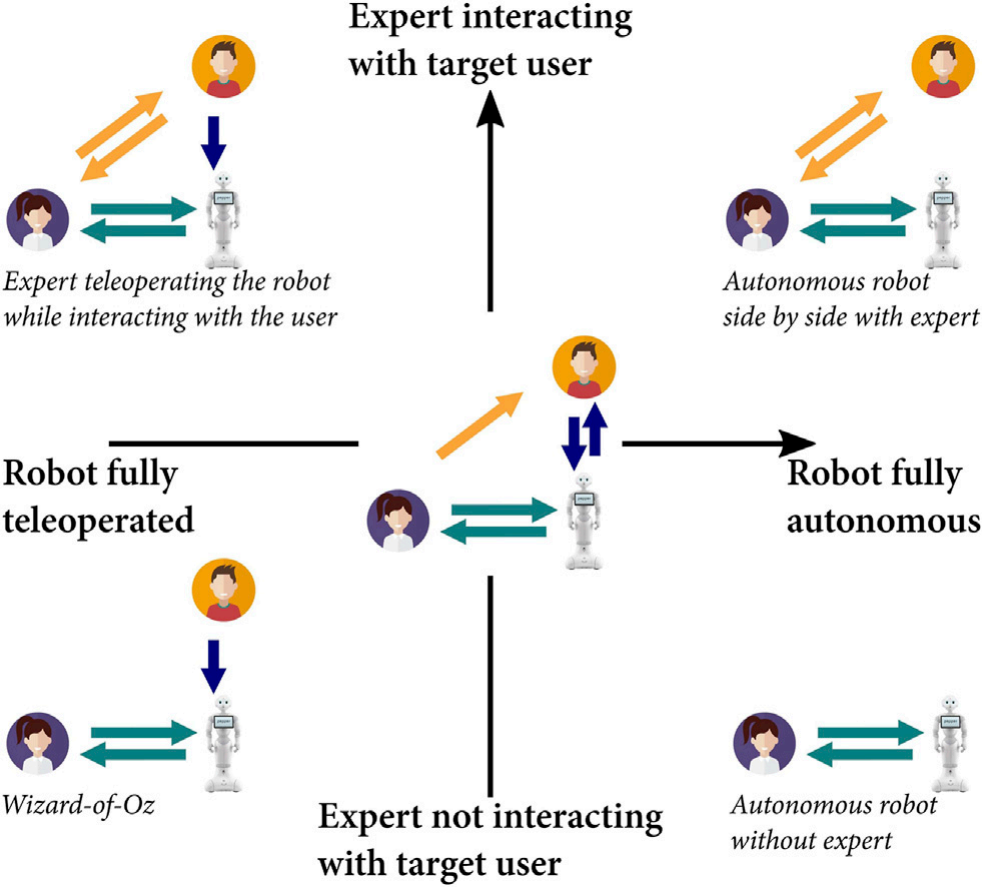
Two-dimensional representation for visualising the different types of long-term expert-robot-user interactions that a social robot might be designed for, all of which LEADOR can facilitate. Note that this is not a discrete space, and LEADOR specifically makes it possible to move along these axes upon real-world deployment. People vector created by studiogstock - www.freepik.com.

We suggest that LEADOR can be used to design robots for a variety of interaction settings, in terms of the required autonomy and the nature of expert-robot-user interactions long-term. We propose two axes to describe the different types of interaction that might be desired, based on the application (Figure 3). A first axis describes the extent to which the domain expert(s) and user(s) are expected to interact long term, as a supplement to the robot-user interaction(s). The second axis reflects the autonomy of the robot, from full supervision (teleoperation) to full autonomy. These two axes are independent as, for example, cases exist where the expert might be continuously interacting with the target users, whilst continuing or not to supervise and/or improve the autonomous behaviour of the robot long term. In addition, these axes do not represent a discrete space, as the teaching interaction element of LEADOR specifically makes it possible to move along either axis at any point during real-world deployment.

The robots developed in our foundational studies demonstrate this flexibility and exist in slightly different spaces on these axes. The educational robot by Senft et al. (2019) is an example of an autonomous robot operating without the expert, and the teaching interaction represented a typical Wizard-of-Oz setup, i.e., there was no interaction. The robot fitness coach by Winkle et al. (2020) is closer to an autonomous robot operating side by side with the domain expert, and the teaching interaction utilised some interactions between the expert and the users (although this was undertaken *outside* of direct teleoperation).

The two foundational studies also demonstrate and evaluate different, complimentary elements of the effectiveness of LEADOR for designing social robots. More specifically, Senft et al. (2019) fundamentally demonstrated the practical feasibility of the teaching interaction for creating appropriate autonomous behaviour. After a teaching phase with 25 children, the robot was deployed autonomously and without expert supervision. It displayed a similar policy to when it was supervised, for example, capturing connections between some events and actions with appropriate timing. However, it was not using a PD approach from the onset, if LEADOR had been applied, then teachers would have been involved more thoroughly in the game design and the interface development.

**FIGURE 4:**
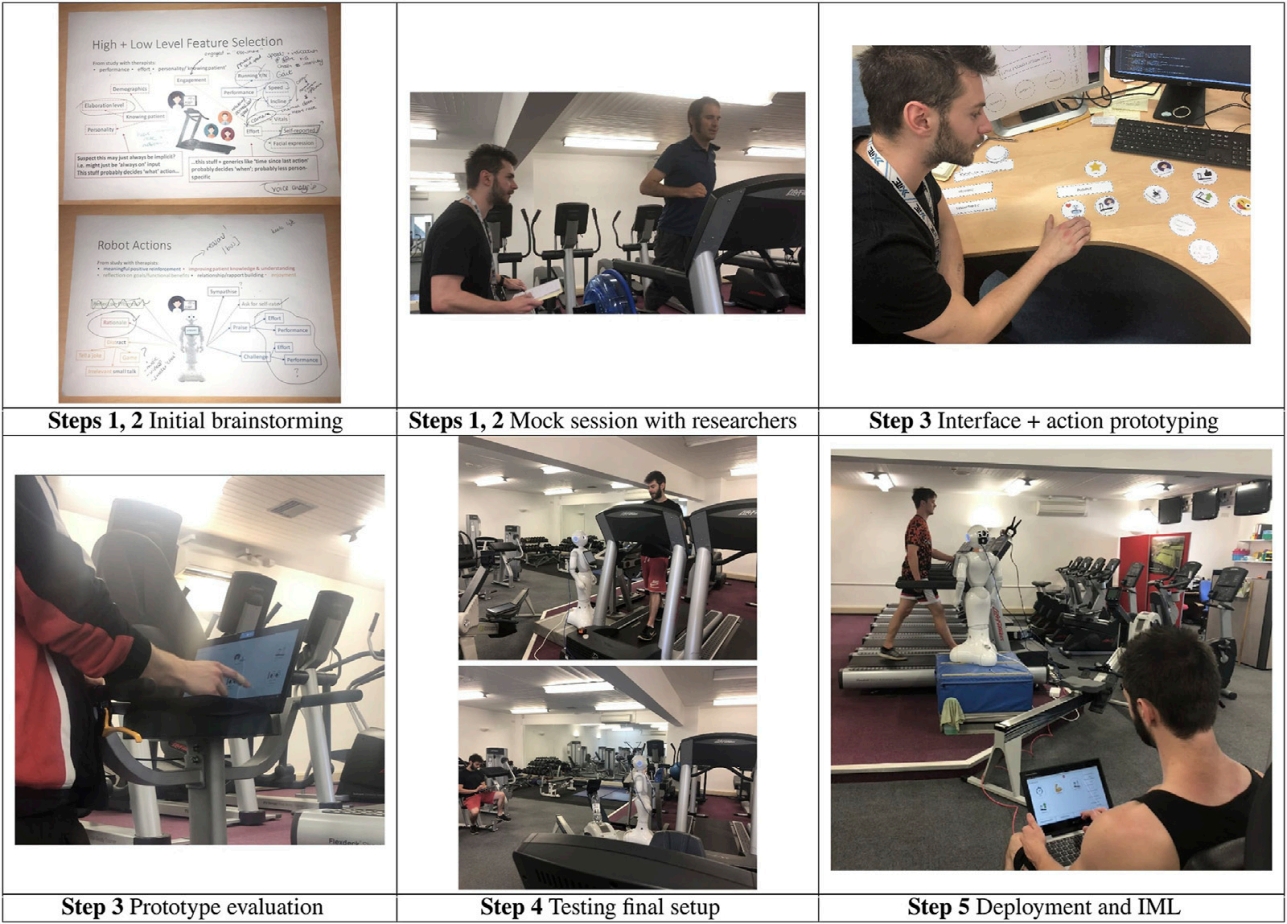
Pictorial representations of the participatory design activities and final teaching setup undertaken in application of our method to the robot fitness coach by Winkle et al. (2020), as per Table 2 with reference to Steps 1–5 of our method as per Figure 1.

Whilst Winkle et al. (2020) again demonstrated similarity between supervised and autonomous behaviour, this work also specifically demonstrated that the teaching interaction resulted in a better autonomous robot than an expert-informed heuristic based alternative. In addition, the work specifically explored to what extent the overall LEADOR could support mutual shaping and influence robot acceptability. To this end, as shown in Figure 4, the significant co-design work undertaken by the domain expert seems likely to have contributed to the high level of ownership he seemed to feel towards the system, and the way in which he conceptualised the robot, throughout, as an independent agentic colleague he was training. When asked whether he perceived Pepper as more of a tool or a colleague, the fitness instructor commented *“It was definitely more of a colleague than a tool […] I like to think her maybe early bugs or quirks definitely gave her a bit more of a personality that maybe I held on to”*. In addition, when evaluating the performance of the robot, the instructor also reflected on the difference between how the robot might behave in comparison to himself: “Pepper’s suggestions might not be what *I* would say in that exact same situation; however, it does not mean that what was said or suggested was wrong”. This gives credibility to the suggestion that LEADOR can be used to create robots that do not simply attempt to imitate or replicate the domain expert directly but instead play a distinct but complimentary role alongside that domain expert in delivering an assistive intervention.

The feedback of the fitness instructor also suggested that the use of the robot did not prevent him from still developing a working relationship with the exercisers or from having a positive impact on their motivation, as he *“did care about their progress and their health”*. This appears to be true on the side of the exerciser, too, because their evaluations suggested they perceived the fitness instructor and the robot as playing distinct but complimentary roles in their undertaking of and engagement with the prescribed exercise programme: *“Pepper was a good instructor and positively motivated my runs. The role of Don [the fitness instructor] assisted this in that having him there meant I could follow the robot’s instructions safe in the knowledge that there was some support there should anything go wrong!”*

In summary, the fitness coach robot example therefore demonstrates the end-to-end PD element of LEADOR, how this seemingly contributes to robot acceptability by both domain experts and target users, and can successfully facilitate meaningful triadic (domain expert-robot-user) interactions in human-centred domains where there might be a desire to reduce domain expert workload without ever removing them from the interaction completely. As such, Winkle et al. (2020) might be seen as a first attempt to fully implement LEADOR ahead of refinement for presentation as a generalisable methodology.

### 5.2 Supporting “Responsible by Design” Robotics

The Foundation for Responsible Robotics (FRR) defines responsible robotics as “the responsible design, development, use, implementation, and regulation of robotics in society”^1^. Concerning research and development, the FRR demonstrates a significant overlap with the goals of mutual shaping and, hence, our goals in proposing LEADOR: “Responsible robotics starts before the robot has been constructed. Ethical decision-making begins in the R&D phase. This includes the kind of research practices that are employed, ensuring that a diverse set of viewpoints are represented in the development of the technology, using methods of development and production that are sustainable, and taking into consideration the impact that the technology will have on all stakeholders to mitigate harm preemptively rather than after the fact.”

A significant number of attempts to more formally define the ethical design and development have taken the form of published principles of AI and robotics ^2^, many of which similarly identify the importance of engaging (non-roboticist) users and domain experts in robot design and evaluation processes. Arguably, one of the more practical resources is the British standard BS8611-2016 *Guide to the Ethical Design and Application of Robots and Robotic Systems* (BSI, 2016), which explicitly identifies ethical risks posed by robots, mitigating strategies and suggested methods for verification and validation. Notably, the standard suggests that a number of the identified ethical hazards might be verified and validated through *expert guidance* and *user validation*. Through LEADOR, such guidance and validation is inherently “built-in” to the design and development process. On the basis of this, we posit that, in supporting a mutual shaping approach to robot development, and specifically by “opening up” robot automation to non-roboticists (such that they can contribute to robot design and automation but also better understand robot capabilities and limitations), LEADOR also represents a concrete implementation of a *responsible robotics* approach and offers a practical way to create social robots with expert guidance and user validation being inherent to the development process. Consequently, whilst the program is evaluated by its designers, these designers are the domain experts and thus the best persons to assess whether the robot behaviour is successful or not.

### 5.3 Future Development

#### 5.3.1 Inclusion of Application Targets in Design, Automation, and Evaluation

A key limitation in both of our foundational works was the lack of including target users during the design processes. This is partly because both of these works are concerned in the development of robots that would be assisting the domain expert practitioners (i.e., a teaching assistant and a fitness instructor), and so, it made sense to focus on working with such experts as co-designers of the system. However, as discussed in the introduction, inclusion of all stakeholders is a key aim of mutual shaping approaches to robot design/development.

A desire to include target users in the design and evaluation of the robot would raise the interesting question of how target users, who are expecting to beneficiaries of the interaction, could design the robot. In a number of situations where the robot is expected to provide support or additional knowledge, including target users in the co-design of the action state, for example, could be either complex or negate the *perception* of the robot as an agent. This also overlaps with discussions in contemporary PD works regarding the “legitimacy” of different PD participants and specifically with the idea of participants *learning* from the researchers as a pre-requisite for becoming such (Ehn, 2008). In the first instance, however, as an obvious extension to LEADOR, target users could certainly be included in preliminary testing of those actions designed with a domain expert.

#### 5.3.2 Alternative Teaching/Learning Interactions

The method presented in this paper focused on a teaching phase where a domain expert teaches the robot how to interact with a target user, with the target user unaware of the extent to which the expert is involved in the robot behaviour. It abstracts away the type of learning used as each situation has different constraints and requires variations on the teaching interaction and learning algorithm. Consequently, LEADOR might not be applicable directly to every situation. Future work should explore the applicability of LEADOR with other interaction designs not explored in our foundational studies and explore combination with other methodologies to extend this applicability whilst maintaining the key tenets regarding expert involvement and *in situ* robot design, teaching, and testing.

Whilst situations such as therapy or education require the expert and target user to be different persons, a large number of other domains relax this constraint. For example, an elderly at home could have a robot carer and teach the robot how to support them in their daily activity. In this case, the target user is the person knowing best their needs and as such would be the perfect expert. LEADOR would be highly applicable to this situation as the target users could be involved early in the design process, help specify the state and action spaces and tools that they would need, and finally teach *in situ* their robot how to interact whilst benefiting from the interaction themselves.

Alternatively, building on the previously noted limitation regarding target user inclusion, applications where the robot is to play more of a *peer* role, rather than an expert authority might be best achieved by having one target user teach the robot how to interact with another target user. This might be particularly appropriate for, e.g., allowing teenagers to automate companion robots that support the mental health of teenagers (Björling and Rose, 2019). This raises a number of interesting research questions regarding how the teaching interaction might impact on the teacher’s (self-)understanding of the application domain, representing another aspect of mutual shaping that could be considered in more detail in future works.

An alternative, exciting teaching interaction is having the teaching phase being open and transparent to the target user. Teaching robots could be similar to how adult teach children to interact, by providing explicit feedback guideline openly in the social environment. This situation raises a number of open questions such as to what extent having the expert providing feedback to the robot could impact the ascribed agency of the robot or how could the target user be included in telling the robot how best to help them. We have good evidence from our work (Winkle et al., 2020) that such open interaction would not “break the illusion” of the robot being an independent (credible) social agent. Further, previous work suggests that robot users value the human developers “behind” the robot, because it is their “genuine intentions” that underlie the social and assistive behaviours of robot (Winkle et al., 2019b). In sensitive application environments such as the previously mentioned teenage mental health support, such openness may indeed be crucial to robot effectiveness and acceptability (Björling et al., 2020).

However, these alternative teacher/learner configurations need to account for the existing practical constraints of using reinforcement learning (RL) in human-robot interaction. Indeed, in the context of HRI, RL faces two main issues: 1) the large number of data points required to improve the policy (which have to come from real-world interaction) and 2) the risks posed by the RL “exploration” in the real HRI, where the RL algorithm might suggest actions that are inappropriate in a given context.

In our two studies, the domain expert also acted as a “gate keeper” for the suggestions of the robot and as a general safety net, able to intervene if the autonomous robot behaviour was inappropriate. Likewise, when applying LEADOR in other scenarios, adequate safeguarding needs to be in place, until further research on RL can provide adequate safety guarantees. Alternatively, the expert could serve early on to help create an initial safe and effective policy by providing a high amount of guidance. Then, in the second phase, the expert could revert only to the “gate keeper” role, working as a safeguard to ensure that the policy of the robot has a minimum efficacy whilst letting the robot self-improve. Finally, when the robot reaches a sufficient expertise in the interaction, it could be left to fine-tune its policy with less supervision.

## 6 Conclusion

In this article, we present LEADOR, a method for end-to-end PD of autonomous social robots that supports a mutual shaping approach to social robotics. This general method is derived from two independent foundational studies and represents a culmination of the experiences of authors working with domain experts in the development of autonomous socially assistive robots. We describe the activities undertaken in those studies to demonstrate how the method has been derived and give tangible examples of how it might be applied. Together, we suggest that these foundational studies also demonstrate both the feasibility and the value of the approach, because both resulted in acceptable, autonomous, and effective socially assistive robots successfully utilised in complex real-world environments.

The first key contribution of LEADOR is to make robot *automation* participatory, such that non-roboticist, domain experts can contribute directly to generating autonomous robot behaviours. This particularity compliments more typical use of PD, e.g., generating the initial robot design guidelines or evaluation robot prototypes. We achieve this expert-led automation by utilising a *teaching interaction*, whereby the domain expert(s) can directly define and refine the autonomous behaviour of the robot through a teaching interface. Both of our foundational studies utilised IML and the SPARC paradigm (Senft et al., 2015), which we suggest is particularly well suited to the overall method goals; therefore, we particularly reflect on this approach and its benefits, challenges, and limitations. However, whilst we refer to this as a teaching interaction, because the domain expert is “teaching” the robot how to behave, our method is agnostic as to the specific technical approach taken (e.g., machine learning and authoring) to facilitate it.

The second key contribution of our LEADOR is to facilitate a mutual shaping approach throughout robot development. This is achieved, first, by the increased domain expert participation in robot automation as described above. In addition, however, our integration of the teaching interaction into real-world robot deployment means that this automation of robot behaviour can actually be informed by and reflect the complex and nuanced realities of the real-world context, capturing the tacit and intuitive responses of the expert to real-world social dynamics. Given that teaching can be re-convened at any time, the method also facilitates the updating of robot behaviours in response to these evolving dynamics or new emerging dynamics, i.e., observation of mutual shaping effects. More generally, the *in situ* robot deployment and expert teaching role maximise the opportunity to identify and understand such mutual shaping effects to better evaluate the overall impact and efficacy of the robot for the proposed application.

In facilitating end-to-end PD and mutual shaping, we also suggest that our method inherently supports responsible robotics, by design. Specifically, it allows for a diverse set of viewpoints to be represented in the development of the technology and for preemptive consideration of the impact that technology will have on stakeholders. Finally, on a practical level, we also suggest our method can better facilitate multi-disciplinary working because it systematically combines PD and technical development such that non-roboticist researchers and stakeholders are no longer excluded from any stage of the development process.

In summary, we suggest that LEADOR is an all-around effective approach for creating socially intelligent robots, as *practical* as it is *responsible* in facilitating the creation of expert-informed, intuitive social behaviours. We identify a number of areas for potential future development, which we hope will be of interest to other roboticists in refining the method further and working further towards democratisation of robot design and development.

## Data Availability

The original contributions presented in the study are included in the article/Supplementary Material, further inquiries can be directed to the corresponding authors.

## Footnotes

1
https://responsiblerobotics.org/

2
http://alanwinfield.blogspot.com/2019/04/an-updated-round-up-of-ethical.html
